# Continuous and repeat metabolic measurements compared between post-cardiothoracic surgery and critical care patients

**DOI:** 10.1186/s12890-023-02657-4

**Published:** 2023-10-16

**Authors:** Koichiro Shinozaki, Pey-Jen Yu, Qiuping Zhou, Hugh A. Cassiere, Stanley John, Daniel M. Rolston, Nidhi Garg, Timmy Li, Jennifer Johnson, Kota Saeki, Taiki Goto, Yu Okuma, Santiago J. Miyara, Kei Hayashida, Tomoaki Aoki, Vanessa K. Wong, Ernesto P. Molmenti, Joshua W. Lampe, Lance B. Becker

**Affiliations:** 1 The Feinstein Institutes for Medical Research, Northwell Health, Manhasset, NY USA; 2 Department of Emergency Medicine, Zucker School of Medicine at Hofstra/Northwell, Hempstead, NY USA; 3 Department of Emergency Medicine, North Shore University Hospital, Manhasset, NY USA; 4 Department of Emergency Medicine, Kindai University Faculty of Medicine, Osaka-Sayama, Osaka, Japan; 5 Department of Cardiothoracic Surgery, North Shore University Hospital, Manhasset, NY USA; 6 Division of Critical Care Medicine of Emergency Medicine, Long Island Jewish Medical Center, New Hyde Park, NY USA; 7 Division of Critical Care Medicine, Department of Medicine, North Shore University Hospital, Manhasset, NY USA; 8 Department of Emergency Medicine, South Shore University Hospital, Bay Shore, NY USA; 9 Nihon Kohden Innovation Center, Cambridge, MA USA; 10 Nihon Kohden Corporation, Tokyo, Japan; 11 Department of Surgery, Medicine, and Pediatrics, Zucker School of Medicine at Hofstra/Northwell, Hempstead, NY USA; 12 ZOLL Medical, Chelmsford, MA USA

**Keywords:** Indirect calorimetry, Oxygen consumption, Carbon dioxide generation, Respiratory quotient, Douglas bag

## Abstract

**Objective:**

Using a system, which accuracy is equivalent to the gold standard Douglas Bag (DB) technique for measuring oxygen consumption (VO_2_), carbon dioxide generation (VCO_2_), and respiratory quotient (RQ), we aimed to continuously measure these metabolic indicators and compare the values between post-cardiothoracic surgery and critical care patients.

**Methods:**

This was a prospective, observational study conducted at a suburban, quaternary care teaching hospital. Age 18 years or older patients who underwent mechanical ventilation were enrolled.

**Results:**

We included 4 post-surgery and 6 critical care patients. Of those, 3 critical care patients died. The longest measurement reached to 12 h and 15 min and 50 cycles of repeat measurements were performed. VO_2_ of the post-surgery patients were 234 ± 14, 262 ± 27, 212 ± 16, and 192 ± 20 mL/min, and those of critical care patients were 122 ± 20, 189 ± 9, 191 ± 7, 191 ± 24, 212 ± 12, and 135 ± 21 mL/min, respectively. The value of VO_2_ was more variable in the post-surgery patients and the range of each patient was 44, 126, 71, and 67, respectively. SOFA scores were higher in non-survivors and there were negative correlations of RQ with SOFA.

**Conclusions:**

We developed an accurate system that enables continuous and repeat measurements of VO_2_, VCO_2_, and RQ. Critical care patients may have less activity in metabolism represented by less variable values of VO_2_ and VCO_2_ over time as compared to those of post-cardiothoracic surgery patients. Additionally, an alteration of these values may mean a systemic distinction of the metabolism of critically ill patients.

**Supplementary Information:**

The online version contains supplementary material available at 10.1186/s12890-023-02657-4.

## Introduction

Oxygen consumption (VO_2_), carbon dioxide generation (VCO_2_), and respiratory quotient (RQ), which is the ratio of VCO_2_ to VO_2_, are important measures of the metabolism in humans [1–3]. These measurements are widely used for patients with a variety of conditions, including post-surgery [1], shock [4], pulmonary and cardiac diseases [5], and critical illness requiring mechanical ventilation [2, 6, 7]. Indirect calorimetry is a non-invasive method, in which VO_2_ and VCO_2_ are measured from concentrations of oxygen and carbon dioxide of inhalation and exhalation [8–10]. Since it is non-invasive, indirect calorimetry has been widely used by clinicians [11, 12] as well as animal studies [13–16]. However, due to the lack of a gold standard, the accuracy has been questioned for nearly 100 years [6, 7, 17, 18].

One of the traditional but standard methods is the Douglas Bag (DB) collection technique, which was first introduced by a physiologist, Claude Gordon Douglas, in 1911 [19]. This technique is still the most reliable gold standard these days [18]. Central focus of the DB method is the accuracy of gas concentrations of inhalation and exhalation. Errors are propagated particularly when a high oxygen concentration gas is used [10]. The DB technique is a method using collection bags that equilibrate concentrations of gases inside a bag. The DB technique exerts higher accuracy if a gas concentration dynamically changes. The concentrations of oxygen and carbon dioxide in exhalation change dynamically during each breath. Therefore, the DB method enables reliable measurements when the accuracy of VO_2_, VCO_2_, and RQ is in need [18]. However, a complexity of the method and lengthy processing time limit the number of measurements that can be performed at bedside.

We developed an automation device that enables continuous and repeat measurements of VO_2_, VCO_2_, and RQ in patients undergoing mechanical ventilation [20]. The accuracy was validated, and it was equivalent to that of the DB technique. To the best of our knowledge, this is the first study that applied repeat measurements of VO_2_, VCO_2_, RQ by using a method equivalent to the DB technique and we will demonstrate the results of continuous metabolic measurements in post-cardiothoracic surgery and critical care patients.

## Materials and methods

### Study design

This was a prospective, observational study conducted at a suburban, quaternary care teaching hospital. Age 18 years or older patients who underwent mechanical ventilation were enrolled. The study protocol was approved by the Institutional Review Board (16–615-North Shore University Hospital). Written informed consent for participation was obtained from patients or next of kin prior to the procedures. If a patient did not hold a capacity for consent or did not have a legally authorized representative or next of kin, the patient was enrolled with waived consent. We excluded patients whose positive end-expiratory pressure (PEEP) setting was higher than 10 cm H_2_O due to an expected gas leak from the mechanical ventilation circuit. Our method and calculation algorithm enabled measurements of VO_2_, VCO_2_, and RQ at a variety range of fraction of inspired oxygen (F_I_O_2_) [21] and therefore, no upper limit was made on an F_I_O_2_ setting of mechanical ventilation.

### Automation system

We previously reported the detail of our methods [20]. This is a unique technique, for which we have devoted most of our time to validate its accuracy and the results can be found in the other report [20]. In brief, ten minutes were given to all patients for acclimating to the apparatus before starting a measurement. For the measurements of VO_2_, VCO_2_, and RQ, inhalation and exhalation were independently analyzed. A commercially available gas analyzer (GF-210R Multi-Gas Module, Nihon Kohden Corporation, Irvine, CA, USA) was used. The gases were sampled from a mechanical ventilator (AVEA® ventilator, CareFusion, San Diego, CA, USA) and the gas concentrations of oxygen and carbon dioxide were measured by the gas analyzer. By using these values, we calculated fraction of expired oxygen (F_E_O_2_) and fraction of expired carbon dioxide (F_E_CO_2_) from the concentrations measured at the exhaust port (FexhO_2_ and FexhCO_2_, respectively). The measurement was performed at bedside, which enabled a real-time and continuous collection of data. A dehumidification device (DHU-1000 Dehumidification Unit, Nihon Kohden Corporation, Tokyo, Japan) was set in conjunction with the gas analyzer: the dehumidification unit was intended for use in dehumidifying a sample gas. We have validated the accuracy of this automation system [20]. The validation included (i) a system response time; (ii) sensor accuracy of gas concentrations; (iii) the performance of mixing chamber; (iv) the calculation algorithm of our automation system; (v) and VO_2_, VCO_2_, and RQ in healthy volunteers compared between the device and the DB methods.

The gas concentrations of inhalation and exhalation were measured alternately, and the duty cycle was 15 min. The gas concentration in inhalation was measured at the first 6 min of the duty cycle. A valve switched the sampling port from inhalation to exhalation and 9 min were given for F_E_O_2_ and F_E_CO_2_ measurements.

### Calculations and analysis

F_I_O_2_, F_E_O_2_, F_I_CO_2_ (fraction of inspired carbon dioxide), F_E_CO_2_, in-circuit humidity and temperature in the dehumidification device, and ambient pressure and temperature around the mechanical ventilator circuit were measured. A minute ventilation volume of exhalation (V_E_), inhalation to exhalation (I:E) ratio, and bias flow setting were recorded from the mechanical ventilator. For the automation system, F_E_O_2_ and F_E_CO_2_ were calculated from the gas concentrations measured at the ventilator exhaust port. The following equations are used in this study:

1 $$\mathrm{R}={\mathrm{V}}_{\mathrm{I}}/{\mathrm{V}}_{\mathrm{E}}$$

2 $${\mathrm{VO}}_{2}={\mathrm{V}}_{\mathrm{I}}\times {\mathrm{F}}_{\mathrm{I}}{\mathrm{O}}_{2}-{\mathrm{V}}_{\mathrm{E}}\times {\mathrm{F}}_{\mathrm{E}}{\mathrm{O}}_{2}$$

3 $${\mathrm{VCO}}_{2}={\mathrm{V}}_{\mathrm{E}}\times {\mathrm{F}}_{\mathrm{E}}{\mathrm{CO}}_{2}-{\mathrm{V}}_{\mathrm{I}}\times {\mathrm{F}}_{\mathrm{I}}{\mathrm{CO}}_{2}$$

where V_I_ is a minute ventilation volume of inhalation and V_E_ is that of exhalation. F_I_CO_2_ is zero since the inhalation gas does not contain CO_2_. RQ, VO_2_, and VCO_2_ are then transformed to equations as follows:

4 $${\mathrm{VO}}_{2}=\left(\mathrm{R}\times {\mathrm{F}}_{\mathrm{I}}{\mathrm{O}}_{2}-{\mathrm{F}}_{\mathrm{E}}{\mathrm{O}}_{2}\right)\times {\mathrm{V}}_{\mathrm{E}}$$

5 $${\mathrm{VCO}}_{2}={\mathrm{F}}_{\mathrm{E}}{\mathrm{CO}}_{2}\times {\mathrm{V}}_{\mathrm{E}}$$

6 $$\mathrm{RQ}={\mathrm{VCO}}_{2}/{\mathrm{VO}}_{2}$$

R is generally derived from the Haldane transformation with the assumption that nitrogen is neither produced nor retained by the body, and that no gases are present other than O_2_, CO_2_, and nitrogen [22]. Because the denominator includes F_I_O_2_ and it goes to zero as F_I_O_2_ increases to 1.0, R increases to infinite number when F_I_O_2_ is 1.0. Therefore, the Haldane transformation limits F_I_O_2_ generally up to 0.6. This is a significant limitation in critical care, in which patients normally require a high level F_I_O_2_. Therefore, we developed a method for measuring R and sought the number of R by using our rodent model [21]. Our results suggested that R was not 1.0 and so V_I_ was not equal to V_E_. While our result is in line with the concept of the Haldane transformation that helps us interpret V_I_ ≠ V_E_, the data from our rodent model also indicates that R may be constant as opposed to dependent on F_I_O_2_ as the calculation from the Haldane transformation outputs. We sought a value of human R calculated from the values obtained from previous human reports [22, 23] and determined it as 1.0097 in this study.

### Statistical analysis

We reported data as mean and standard deviation and descriptive statistics were used. The values were reported as standard temperature and pressure and dry (STPD). Spearman’s rho and correlation coefficients were calculated, and Mann–Whitney U tests were used for comparison between two groups. Generalized linear mixed model, in which covariates include survival outcomes and number of measurements, was used to estimate the effects on continuous and repeat measurements. Prism for Mac version 9 (GraphPad Software, San Diego, CA) and SPSS version 27 (IBM, Armonk, NY) were used for statistical analysis, and P values less than 0.05 was considered statistically significant.

## Results

Data from 10 patients were analyzed in this study. Table 1 shows baseline characteristics of the study subjects. Out of 10 subjects, 4 were patients post cardiothoracic surgery, 5 were patients in the intensive care units (ICU), and 1 was in the emergency room (ER). Sequential organ failure assessment (SOFA) score, blood lactate level (mmol/L), and P/F ratio (arterial partial oxygen pressure to F_I_O_2_) were obtained at ICU admission. Female patients had significantly lower weights and heights than male (*p* < 0.05 and *p* < 0.05, respectively). None of the post-cardiothoracic surgery patients had major adverse events nor died. This study included 6 critical care patients, whose metabolic measurements were performed in the ER or ICU, and 3 were died.

**Table 1:** Characteristics of study subjects and fraction of inspired oxygen when starting the measurements

Subject	Site	Age	Gender	Weight (kg)	Height (cm)	BMI (kg/m^2^)	Diagnosis	SOFA	Lactate (mmol/L)	F_I_O_2_	Ve (L/min)	P/F ratio	PEEP (cmH2O)	Discharge Outcome
1	CTICU	52	Male	76	173	25	CAD	3	2	1.00	6.9	233	5	Alive
2	CTICU	63	Male	106	185	31	CAD	3	1.7	1.00	5.1	155	5	Alive
3	CTICU	72	Male	82	167	29	Aortic Aneurysm, Aortic Valve Stenosis	5	1.7	1.00	4.3	207	5	Alive
4	CTICU	62	Male	72	175	24	CAD	7	1.9	1.00	9.0	296	10	Alive
5	ER	85	Female	35	152	15	Aspiration Pneumonia, Respiratory Arrest	6	7.1	1.00	6.4	277	5	Death
6	MICU	76	Male	63	178	20	Multiple Myeloma, Renal Failure	9	1.4	0.60	8.2	392	5	Death
7	MICU	62	Male	84	170	29	Cirrhosis, Pulmonary Fibrosis, Respiratory Failure	12	2.9	1.00	7.7	68	10	Death
8	MICU	92	Male	69	178	22	Pneumonia, Vocal Cord Paralysis, Respiratory Failure	4	1.1	1.00	7.6	260	5	Alive
9	MICU	70	Female	55	165	20	Airway Compromise, Aspiration Pneumonia	0	1	0.30	7.1	403	5	Alive
10	MICU	71	Female	62	152	27	COPD exacerbation, Hypercapnia	0	5.7	0.25	4.1	633	5	Alive

Sequential Organ Failure Assessment (SOFA) score, blood lactate level, and arterial partial oxygen pressure / F_I_O_2_ (P/F) ratio were calculated from the values at ICU admission. Ve is a minute ventilation volume measured at the exhaust port and the values are expressed as BTPS (body temperature, pressure, water vapor saturated)

*CTICU* stands for cardiothoracic intensive care unit, *ER* emergency room, *MICU* medical intensive care unit, *CAD* coronary artery disease, *COPD* chronic obstructive pulmonary disease

### Initial metabolic values

Table 2 demonstrates values of initial metabolic measurements. SOFA score negatively correlated with RQ (- 0.657, *p* < 0.05). Initial blood lactate levels negatively correlated with VCO_2_ (-0.671, *p* < 0.05) but adjusting it by body weight rejected the significance. F_I_O_2_ negatively correlated with RQ (-0.696, *p* < 0.05) (Supplement).

**Table 2:** Metabolic values

Subject	Initial Values					Repeat Measurements					
	VO2 (ml/min)	VCO2 (ml/min)	VO2/BW (ml/kg/min)	VCO2/BW (ml/kg/min)	RQ	# of samples	Length (min)	VO2 (ml/min)	VCO2 (ml/min)	VO2/BW (ml/kg/min)	VCO2/BW (ml/kg/min)
1	249	144	3.28	1.89	.58	17	270	234 ± 14	160 ± 9	3.08 ± 0.18	2.11 ± 0.12
2	202	133	1.91	1.25	.66	33	540	262 ± 27	184 ± 20	2.47 ± 0.25	1.74 ± 0.19
3	158	106	1.93	1.29	.67	24	390	212 ± 16	149 ± 14	2.58 ± 0.20	1.82 ± 0.17
4	238	139	3.31	1.93	.58	26	435	192 ± 20	131 ± 6	2.67 ± 0.28	1.82 ± 0.08
5	132	63	3.77	1.80	.48	5	75	122 ± 20	58 ± 9	3.49 ± 0.57	1.65 ± 0.26
6	210	133	3.33	2.11	.63	48	735	189 ± 9	133 ± 5	3.00 ± 0.14	2.11 ± 0.08
7	181	96	2.15	1.14	.53	14	300	191 ± 7	107 ± 5	2.27 ± 0.08	1.27 ± 0.06
8	236	133	3.42	1.93	.56	10	210	191 ± 24	129 ± 3	2.77 ± 0.35	1.87 ± 0.04
9	213	149	3.87	2.71	.70	18	330	212 ± 12	155 ± 7	3.85 ± 0.22	2.82 ± 0.13
10	117	92	1.89	1.48	.78	17	300	135 ± 21	103 ± 16	2.18 ± 0.34	1.66 ± 0.26

The values of repeat measurements are expressed as means and standard deviations

*BW* stands for body weight, *RQ* respiratory quotient

### Continuous and repeat metabolic measurements

**Fig. 1:**
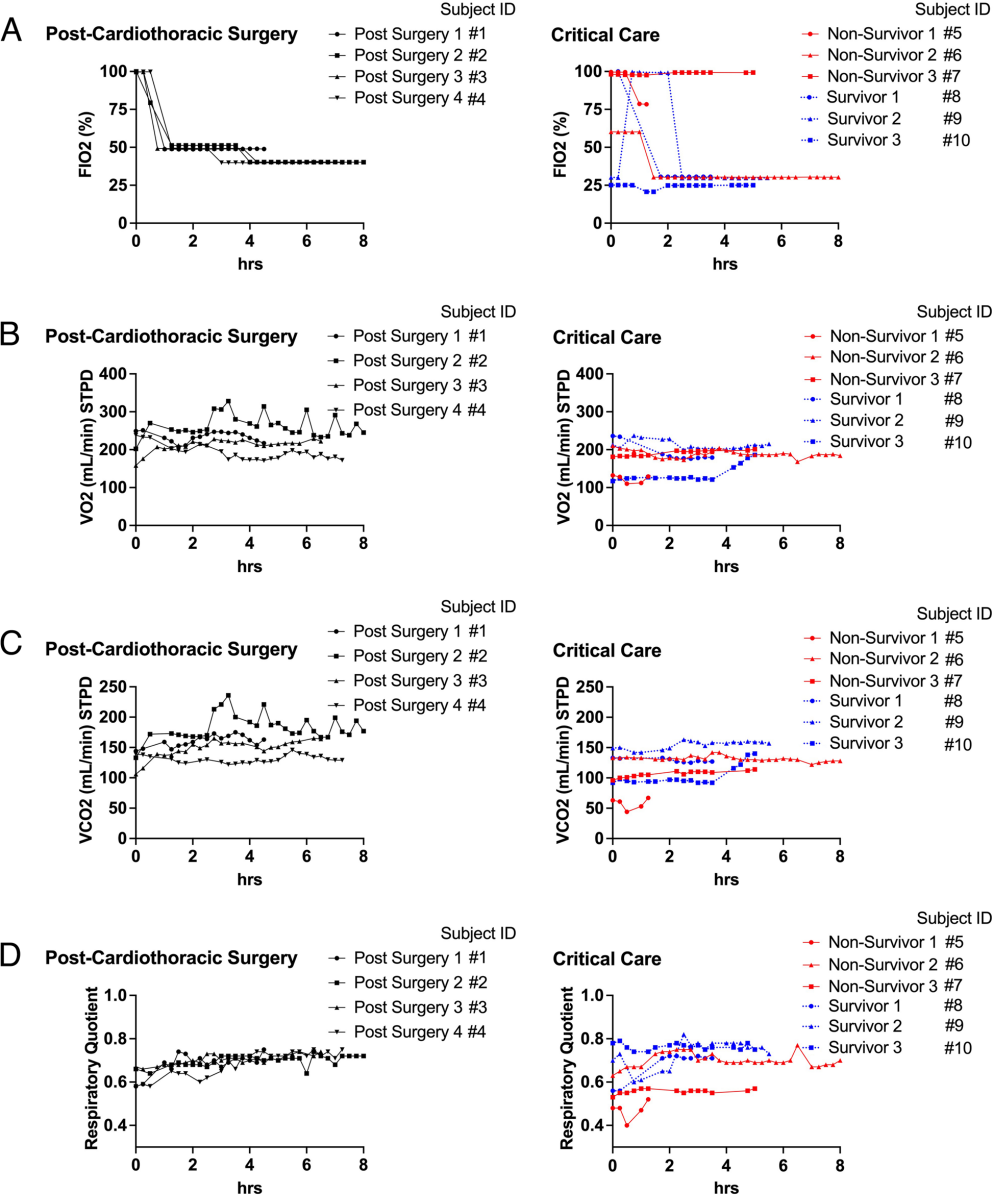
Continuous and Repeat Metabolic Measurements. The measurements were started from the admission to the cardiothoracic intensive care unit for post-surgical patients. For critical care patients, the measurements were started from the earliest possible timing after enrollment. **A** Fraction of inspired oxygen (FIO2). **B** VO2 at standard temperature and pressure and dry (STPD). C VCO2 at STPD. D RQ. *n* = 4 for post-surgery, *n* = 6 for critical care patients. Subjects IDs are corresponding to those in Table 1

We performed continuous and repeat metabolic measurements by the automation device and Fig. 1 and Table 2 show the results. The minimum number of measurements were 5 cycles for 75 min occurred in ER and the longest measurement lasted for 12 h and 15 min and 50 cycles of repeat measurements were performed. Figure 1 A shows the trend of F_I_O_2_ over time. There was a downward trend of F_I_O_2_ in the post-surgery patients because all patients were weaned off from mechanical ventilation during the post-day 1 of surgery. In contrast, there was no consistent pattern of F_I_O_2_ in the critical care patients.

The mean VO_2_ in the 4 post-surgery patients were 234 ± 14, 262 ± 27, 212 ± 16, and 192 ± 20 mL/min, respectively. Those in the critical care patients were 122 ± 20, 189 ± 9, 191 ± 7, 191 ± 24, 212 ± 12, and 135 ± 21 mL/min, respectively. The value of VO_2_ was variable in the post-surgery patients and the range was 44, 126, 71, and 67, respectively. These values seemed more variable than those (22, 42, 20, 60, 38, 69) of critical care patients. The range of non-survivors (22, 42, 20) appeared to be less than those (60, 38, 69) of survivors.

**Fig. 2:**
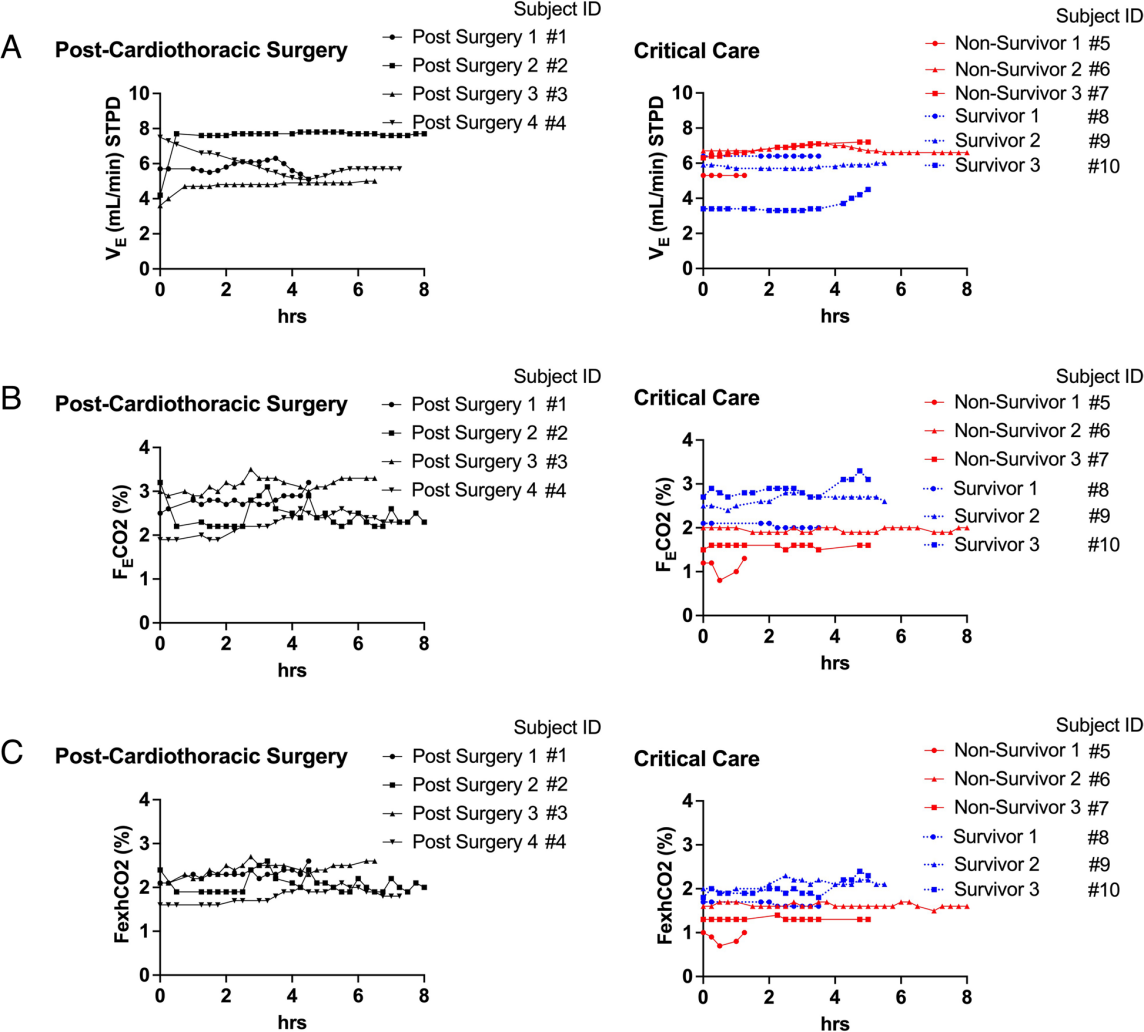
Ventilation and Carbon Dioxide Levels. **A** Minute ventilation volume (SPTD) measured at the exhaust port. **B** Fraction of expired carbon dioxide (FECO2). C Fraction of expired carbon dioxide measured at the exhaust port. *n* = 4 for post-surgery, *n* = 6 for critical care patients. Subjects IDs are corresponding to those in Table 1

The mean VCO_2_ in the 4 post-surgery patients were 160 ± 9, 184 ± 20, 149 ± 14, and 131 ± 6 mL/min, respectively. Those in critical care patients were 58 ± 9, 133 ± 5, 107 ± 5, 129 ± 3, 155 ± 7, and 103 ± 16 mL/min, respectively. The value of VCO_2_ was variable in 4 post-surgery patients and the range was 31, 103, 59, and 24, respectively. These values seemed more variable than those (23, 21, 18, 8, 21, 48) of critical care patients. Figure 2 shows the result of detailed analysis on VCO_2_. According to Eq. (5), VCO_2_ is derived from V_E_ and F_E_CO_2_. The higher VO_2_ found in post-surgery patients seemed associated with both variables. Non-survivors had lower F_E_CO_2_ than survivors and the same trend was found by the plots in fraction of CO_2_ at exhaust port of the mechanical ventilator (FexhCO_2_). Generalized linear mixed model identified the effects of outcome on VO_2_ (*p* < 0.05), VCO_2_ (*p* < 0.01), and VCO_2_ per weight (*p* < 0.01), but not on VO_2_ per weight.

The mean RQ in the 4 post-surgery patients were 0.69 ± 0.05, 0.70 ± 0.02, 0.70 ± 0.02, 0.69 ± 0.06 respectively. Those in critical care patients were 0.47 ± 0.04, 0.71 ± 0.03, 0.56 ± 0.01, 0.68 ± 0.06, 0.73 ± 0.06, 0.76 ± 0.01, respectively. The range of RQ of the post-surgery patients were 0.17, 0.10, 0.08, 0.17 and those of the critical care patients were 0.12, 0.14, 0.04, 0.16, 0.22, and 0.05, respectively.

## Discussion

We developed a system for measuring F_I_O_2_, F_E_O_2_, and F_E_CO_2_ in humans undergoing mechanical ventilation. These gas concentrations are critical components when seeking accurate VO_2_, VCO_2_, and RQ. The accuracy of our system was equivalent to the gold standard DB collection technique [20]. Our automation system allows for continuous and repeat measurements, to which the DB technique has a significant limitation due to the complexity of its procedure. By using our automation system, we successfully collected continuous values of VO_2_, VCO_2_, and RQ in post-surgical and critical care patients.

The volume ratio of inhalation to exhalation defined as R in this study is an essential component of calculating VO_2_. However, there is a significant technical difficulty of measuring the small differences between V_I_ and V_E_. Therefore, V_I_ is commonly calculated by using the Haldane transformation, which limits the use of F_I_O_2_ up to 0.6. Assuming V_I_ equals V_E_ and ignoring this small difference eliminate the concern. However, failure to account for this small difference can erroneously decrease VO_2_ by 17% [10], if V_I_ is actually not equal to V_E_, and the error propagates at higher F_I_O_2_ levels. The adequacy of the Haldane transformation [22] supports that V_I_ is not equal to V_E_.

We have developed a method for measuring the small differences between V_I_ and V_E_ [21]. Our results from the rodent model showed that R ranged from 1.0081 ± 0.0017 to 1.0092 ± 0.0029 at F_I_O_2_ 0.3 and 1.0, respectively, indicating that V_I_ is not equal to V_E_ (R ≠ 1.0000). While our results were in line with the concept of the Haldane transformation supporting the idea of V_I_ ≠ V_E_, our data revealed that R is an independent value from F_I_O_2_ as opposed to that calculated by the Haldane transformation, which includes F_I_O_2_ in its equation. We sought a value of human R calculated from the values obtained from previous human reports [22, 23] and determined it as 1.0097 in this study. These data justify the use of R = 1.0097 to all calculations that include various F_I_O_2_ levels that depend on patient’s condition.

Our automation system allows for continuous and repeat measurements of VO_2_, VCO_2_, and RQ. The data of 10 patients were evaluated in this study. Post-surgical patients had higher VO_2_ and VCO_2_ and appeared to have more variable numbers as compared to those of critical care patients. It is inferred that the post-surgical patients had more active metabolism than critical care patients, which might be attributed to purposefully minimized sedatives to wean off the patients from mechanical ventilation. The greater variability of metabolic values argues the unmet need of continuous measurements. In other words, one point measurement such as the traditional DB technique, even though it is accurate and reliable, may not be sufficient to identify an important signal that varies over time. We also found that non-survivors appeared to have low F_E_CO_2_. This is inferred that patients who are critically ill may have less activity in metabolism.

Differences in study values were observed between survivors and non-survivors at the beginning of the measurements. This suggests that significant systemic distinctions occurred at the starting period of our measurements and those could induce changes in the values. Indeed, SOFA scores were higher in non-survivors and there was a negative correlation of VCO_2_ with the initial value of blood lactate, while there was no statistically significant correlation between SOFA and VCO_2_. Both SOFA scores and blood lactate are known to indicate the severity of diseases but the difference of the correlation patters of VCO_2_ with these values may be led by a mechanistic response. Moreover, our study subjects include female patients, whose weights and heights were significantly lower than male. Adjusting VCO_2_ by weight rejected the correlation of VCO_2_ with blood lactate, meaning it might be confounded by the body size. On the other hand, RQ is not affected by the body size as its calculation cancels the effect of body weight. Indeed, there were negative correlations of RQ with SOFA and FIO_2_. We found that high FIO_2_ leads to lower RQ due to the increase in VO_2_ [20]. Therefore, this finding might mean that the lowered RQ could be led by the distinctions of the patients or high FIO2 or both. However, due to the small number of our study population, we could not draw a conclusion. Further studies may warrant the investigation of these mechanisms.

This study is subject to several limitations. The number of samples, first and foremost, is limited in this study owing to the nature of explanatory design of our current work. Second, the study lacks a mechanistic information. More detailed information such as the dose of inotropes, vasoactive inotropic score, and nutrition would help with identifying specific characteristics or reasons that alter VO_2_, VCO_2_, or RQ in the ICU settings. Particularly because nutrition [3] and/or disease conditions [24, 25] may alter RQ, more focused information on these variables needs to be investigated. The automation system aids clinicians to collect more information on metabolism of patients. Moreover, our method enables the gas concentration measurements that are equivalent to the gold standard DB method. Therefore, we expect to see more metabolic data from patients in the future.

## Conclusions

We developed an automation system that enables repeat measurements of VO_2_, VCO_2_, and RQ. Critical care patients may have less variability of VO_2_ and VCO_2_ as compared to the post-cardiothoracic surgery patients.

### Supplementary Information

**Additional file 1:** **Table S1.** Differences of values between post-surgery and critical care patients. **Table S2.** Differences of values between survivors and non-survivors. **Table S3.** Differences of values between male and female patients. **Table S4.** Correlation between study values. **Table S5.** Mechanical ventilator settings.

## Data Availability

The datasets generated and/or analyzed during the current study are not publicly available due the current sponsorship made with Nihon Kohden Corporation but are available from the corresponding author on reasonable request.
