# Supplementary material for: Continuous and repeat metabolic measurements compared between post-cardiothoracic surgery and critical care patients
Source: BMC Pulm Med. 2023 Oct 16;23:390. doi: 10.1186/s12890-023-02657-4 (PMC10577926; doi:10.1186/s12890-023-02657-4)
Supplement: Supplementary file 1 — Additional file 1: Table S1. Differences of values between post-surgery and critical care patients. Table S2. Differences of values between survivors and non-survivors. Table S3. Differences of values between male and female patients. Table S4. Correlation between study values. Table S5. Mechanical ventilator settings. [file 12890_2023_2657_MOESM1_ESM.docx]

Title Page

Continuous and repeat metabolic measurements compared between post-cardiothoracic surgery and critical care patients.

Authors: Koichiro Shinozaki^1,5,6,12^, MD, PhD; Pey-Jen Yu^2^, MD; Qiuping Zhou^3^, DO; Hugh A. Cassiere^4^, MD; Stanley John^4^, MHA, RT; Daniel M. Rolston^5,6^, MD, MSHPM; Nidhi Garg^5,7^, MD; Timmy Li^5^, PhD; Jennifer Johnson^6^, BA; Kota Saeki^1,8,^ MEng; Taiki Goto^9^, MBE, Yu Okuma^1^, MD, PhD; Santiago J. Miyara^1^, MD, PhD; Kei Hayashida^1^, MD, PhD; Tomoaki Aoki^1^, MD, PhD; Vanessa K. Wong^1^, BS; Ernesto P. Molmenti^1,10^, MD; Joshua W. Lampe, PhD^1,11^; and Lance B. Becker^1,5,6^, MD, FAHA

Corresponding to:

Koichiro Shinozaki

Department of Emergency Medicine

Zucker School of Medicine at Hofstra/Northwell

The Feinstein Institutes for Medical Research

350 Community Dr. Manhasset, NY 11030

Telephone: (516) 562-2943

E-mail: shino@gk9.so-net.ne.jp

RESULTS OF CORRELATION ANALYSIS

Table S1. Differences of values between post-surgery and critical care patients.

Table S2. Differences of values between survivors and non-survivors.

Table S3. Differences of values between male and female patients.

Table S4. Correlation between study values.

Correlation is significant at 0.01 level (2-tailed)**; 0.05 level (2-tailed)*; the data includes 10 study patients.

Table S5. Mechanical ventilator settings.

| **Subject ID** | **FiO2 level** | **PEEP**  **[cm H_2_O]** | **Room air pressure [hPa]** | **Room temp [deg.C]** | **Ve (BTPS) [L/min]** | **Ve (ATPD) [L/min]** | **Ve (STPD) [L/min]** | **I/E** | **Bias flow setting(L/m)** |
| --- | --- | --- | --- | --- | --- | --- | --- | --- | --- |
| 1 | 100% | 5 | 1016 | 23.1 | 6.9 | 6.2 | 5.7 | 0.50 | 2.0 |
| 2 | 100% | 5 | 1013 | 22.1 | 5.1 | 4.6 | 4.2 | 0.38 | 2.0 |
| 3 | 100% | 5 | 1012 | 21.5 | 4.3 | 3.8 | 3.6 | 0.28 | 2.0 |
| 4 | 100% | 10 | 1016 | 22.5 | 9.0 | 8.1 | 7.5 | 0.29 | 2.0 |
| 5 | 100% | 5 | 1018 | 24.0 | 6.4 | 5.8 | 5.3 | 0.50 | 2.0 |
| 6 | 60% | 5 | 1007 | 22.3 | 8.2 | 7.3 | 6.7 | 0.30 | 2.0 |
| 7 | 100% | 10 | 1006 | 21.9 | 7.7 | 6.9 | 6.3 | 0.33 | 2.0 |
| 8 | 100% | 5 | 1027 | 23.3 | 7.6 | 6.8 | 6.4 | 0.23 | 2.0 |
| 9 | 30% | 5 | 1021 | 21.7 | 7.1 | 6.3 | 5.9 | 0.23 | 2.0 |
| 10 | 25% | 5 | 1015 | 21.6 | 4.1 | 3.7 | 3.4 | 0.14 | 2.0 |

Ve stands for minute ventilation measured at the exhaust port; I/E inspiration and expiration ratio.
